# A Wafer Pre-Alignment Algorithm Based on Weighted Fourier Series Fitting of Circles and Least Squares Fitting of Circles

**DOI:** 10.3390/mi14050956

**Published:** 2023-04-27

**Authors:** Jingsong Chen, Zhou Lan, Cheng Xue, Jun Lan, Zhenghao Liu, Yong Yang

**Affiliations:** 1 National Key Laboratory of Optical Field Manipulation Science and Technology, Chinese Academy of Sciences, Chengdu 610209, China; chenjinsong21@mails.ucas.ac.cn (J.C.);; 2 State Key Laboratory of Optical Technologies on Nano-Fabrication and Micro-Engineering, Institute of Optics and Electronics, Chinese Academy of Sciences, Chengdu 610209, China; 3 Institute of Optics and Electronics, Chinese Academy of Sciences, Chengdu 610209, China; 4 University of Chinese Academy of Sciences, Beijing 100000, China

**Keywords:** wafer pre-alignment, rotary scanning edge detection, weighted Fourier series fitting of circles method, the least squares fitting of circles method

## Abstract

The wafer pre-aligner is a crucial component in the lithography process to correct the wafer center and notch orientation. To improve the precision and the efficiency of pre-alignment, a new method to calibrate the center and the orientation of a wafer based on the weighted Fourier series fitting of circles (WFC) method and the least squares fitting of circles (LSC) method, respectively, is proposed. The WFC method effectively suppressed the influence of the outliers and had high stability compared with the LSC method when fitted to the center of the circle. While the weight matrix degenerated to the identity matrix, the WFC method degenerated into the Fourier series fitting of circles (FC) method. The fitting efficiency of the FC method is 28% higher than that of the LSC method, and the fitting accuracy of the center of the FC method is the same as that of the LSC method. In addition, the WFC method and the FC method perform better than the LSC method in radius fitting. The pre-alignment simulation results showed that the absolute position accuracy of the wafer was ±2 µm, the absolute direction accuracy was 0.01°, and the total calculation time was less than 3.3 s in our platform.

## 1. Introduction

In the manufacture of integrated circuits (IC), the key to making the device smaller and lowering the cost of a single transistor is the continuous improvement of the resolution and efficiency of the photolithography system [1]. The equipment front-end modules (EFEM) is an important subcomponent of the lithography system, which is mainly responsible for transporting the wafers in the cartridge to the work stage. This step requires the wafer to maintain a high positioning accuracy. However, when wafers are stored and transported, the accumulated errors will inevitably reduce their position accuracy [2]. Therefore, it is necessary to calibrate the center and the orientation of the wafer by using the wafer pre-alignment device. The wafer pre-alignment device is the only calibration device in EFEM, its efficiency determines the production, and its accuracy determines the pass rate [3].

There are three steps in wafer pre-alignment: edge position acquisitioning, wafer positioning, and wafer calibrating. At present, the methods of obtaining the edge position of the wafer include machine vision recognition [4,5,6] and rotary scanning edge recognition [7,8,9]. The wafer with a diameter of 300 mm or above is difficult to quickly obtain the edge image with micron-scale accuracy at one time through machine vision due to the limitation of camera resolution and long post-processing time consumption [10]. Xu et al. proposed the Fourier transform algorithm for orientating and the least squares fitting of circles (LSC) method for positioning to solve this problem [11]. Specially, they took an image of the cell arrangement firstly. Then, they applied the Fourier transform algorithm to the image to calculate the orientation of the wafer. However, wafers need to be lithographed at least once before they have cells. We cannot use this method to pre-align a wafer that has not been patterned. In addition, in order to position by only one image, they firstly obtained the local edge of the wafer, then the LSC method was used for fitting. However, the LSC method is poor for local arc fitting, which limits the accuracy of the method [12].

For those reasons, the rotating edge scanning detection method has become the mainstream method of wafer edge acquisition. Many researchers combine the CCD linear array sensor or other optical measuring elements with rotating platforms to construct test platforms, which is applied to collect the distance $\left(d_{i}\right)$ between the wafer edge and the sensor and the rotation angle $\left(\theta_{i}\right)$ of the rotating platform [7,13]. Then, these data are used to fit the center of the wafer. The way these data are used is a key factor in determining efficiency and fitting accuracy. The mean estimation algorithm (ME) is the simplest fitting method. However, this method is easily affected by the uniformity of points, noise, and notch. Subsequently, researchers proposed an optimization algorithm to fit the data. We constructed the equation based on the observed information while fitting. The optimal estimation criterion was used to approximate the parameters of the equation, and the relative best curve was obtained. In previous studies, most scholars directly used the circle equation as the wafer edge fitting equation, then they used the least square method to approximate the parameters [11,14,15]. A similar method is also widely used in triboelectric nanogenerator monitoring image processing [16,17]. However, they need to convert the data (d,θ) into cartesian coordinates while the LSC method is used to fit the wafer edge [18]. The process of data conversion is time-consuming, and it will add new errors, which reduces computational efficiency and fitting accuracy, respectively. In addition, the LSC method is used to find the global optimal solution, which is easily affected by outliers. In order to not convert the data (d,θ), Huang et al. proposed to use the centroid estimation algorithm (CE) to positioning and orientation [9]. However, this method will be affected by the notch when fitting the center of the circle. In addition, when fitting the notch, the notch needs to be subdivision sampled, which is inefficient.

To solve these problems, the weighted Fourier series fitting circle method (WFC) is proposed in this paper for positioning, and the LSC algorithm is proposed for orientating. The simulation results show that when adding Gaussian noise and outliers to the edge points, the WFC method can suppress outliers better and has higher stability than the conventional LSC method for wafer positioning. When the weighted matrix degenerates to the identity matrix, the WFC method is changed into the Fourier series fitting circle method (FC). On the other hand, the LSC method or FC method are used to fit the center of the circle, and the simulation results showed that the two have similar performance in the center of the circle fitting. However, the efficiency of the FC method is improved. Additionally, the radius fitting error of the WFC method is only one-third of the LSC method whether the weighted matrix is degenerated or not.

## 2. Circle Fitting Method

**Figure 1:**
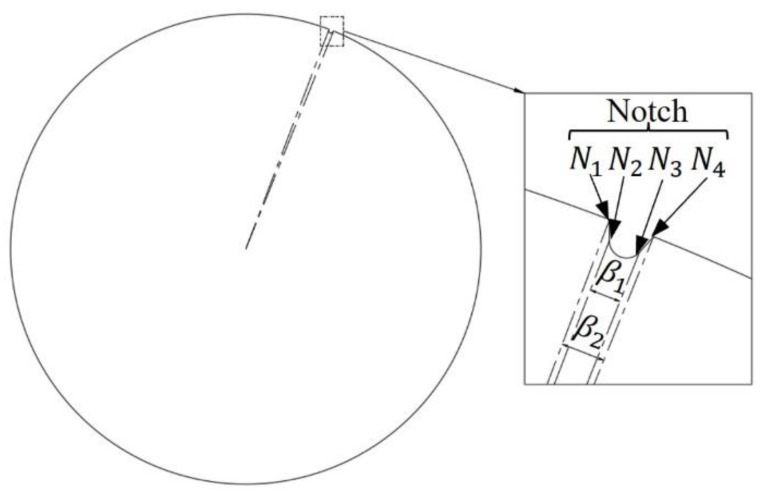
300 mm Wafer.

In general, wafers are manufactured according to the SEMI standard. The shape of the 300 mm wafer is shown in Figure 1. The wafer edge includes circle edge and notch $N_{1}N_{4}$. The $N_{1}N_{2}$ and $N_{3}N_{4}$ segments of notch are straight lines, and the $N_{2}N_{3}$ segment is a short arc. The central angle $\beta_{2}$ of the notch is 1.04°. The central angle $\beta_{1}$ of the $N_{2}N_{3}$ arc is 0.49°. When the wafer is pre-aligned, the circle edge needs to be fitted to find the center of the wafer. Then, the $N_{2}N_{3}$ segment is fitted to find the orientation of the wafer.

**Figure 2:**
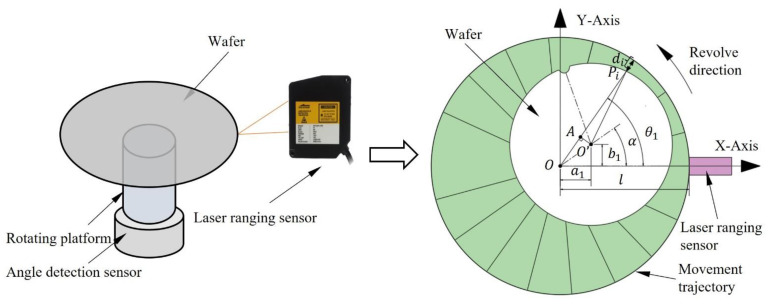
Rotating scanning edge detection method.

The first step of edge fitting is collecting edge data. We use the rotary scanning edge detection method to detect the wafer. The measurement accuracy of this method is the submicron scale. During the rotating scanning edge detection, the rotating platform drives the wafer to rotate. Meanwhile, the laser ranging sensor measures the distance $\left(d_{i}\right)$ from the wafer edge to the sensor, and the angle detection sensor measures the rotation angle $\left(\theta_{i}\right)$ of the wafer in real time. It can also be represented as a relative rotation of the sensor around the wafer. The principle of the wafer edge rotation scanning is shown in Figure 2. Where O is the center of rotation, $O^{’}$ is the center of the wafer. The coordinate system XOY is always fixed, α is the angle of the wafer center in the coordinate system XOY at the initial position, and $\left(a_{1},b_{1}\right)$ is the initial position of the center of the wafer in the coordinate system XOY. In the experiment, the rotating platform drives the wafer rotating around the rotation center O. Additionally, the edge point can be expressed by the point ($d_{i},\theta_{i}$). While the rotating platform revolved once, n points can be obtained.

### 2.1. Fitting the Center of the Wafer by WFC

Generally, scholars constructed circular equations based on wafer shape, and then used the least square method to fit. Therefore, they need to convert the $\left(d_{i},\theta_{i}\right)$ vector into XOY cartesian coordinates to calculate [18]. Obviously, if the model is fitting $\left(d_{i},\theta_{i}\right)$ directly, the operation time can be reduced significantly, and the efficiency can be improved.

In Figure 2, if we unfold the green area, we obtain a curve. Obviously, this curve is a periodic curve, which can be expressed by the Fourier series [19]. Therefore, we can analyze the geometrical characteristics with the detection of wafer edge points and obtain the expression equation in the form of a Fourier series. At the same time, we also need to find the physical meaning corresponding to the coefficient of the Fourier series.

To fit the Fourier series directly using the points, we assume that the offset distance between the center O′ of the wafer and the rotation center O is m. The distance between any edge point $p_{i}$ and the rotation center O can be expressed as ${OP}_{i}$. It can be calculated as

(1) $$OP_{i}=OA+AP_{i}=m\cos(\theta_{i}-\alpha )+\sqrt{R^{2}-m^{2}\sin^{2}(\theta_{i}-\alpha )}$$

Although the center of the wafer will be offset when the wafer is transported, the offset is generally less than 10 mm. Additionally, the radius of a 300 mm wafer is 150 mm. Where m≪R and $\sin^{2}\left(\theta_{i}-\alpha \right)\leq 1$. Therefore, Formula (1) can be approximately simplified as

(2) $$OP_{i}=R+m\cos(\theta_{i}-\alpha )$$

Furthermore, the distance of the laser ranging sensor and the center of rotation O is a constant l, so the theoretical distance $D_{i}$ from the wafer edge to the sensor can be expressed as

(3) $$D_{i}=l-R-m\cos\alpha \cos\theta_{i}-m\sin\alpha \sin\theta_{i}$$

In Formula (3), $\left(l-R\right)$, mcosα and msinα are constants. Additionally, mcosα is the deviation in the X direction, msinα is the deviation in the Y direction and ${l-a}_{0}$ is the radius of the fitting circle. If there is an ideal curve that perfectly fits the data, $D_{i}$ is the fitting value. While we fit the data, we need to compare the difference between $D_{i}$ and the actual value $d_{i}$. Therefore, the error *V_i_* between fitting point and measuring point can be written in the form of the Fourier series

(4) $$V_{i}=D_{i}-d_{i}=a_{0}-a_{1}\cos\theta_{i}-b_{1}\sin\theta_{i}-d_{i}$$

where $a_{1}=m\cos\alpha$, $b_{1}=m\sin\alpha$, and $a_{0}=l-R$.

Then, the error formula of each fitting point can be further expressed as

(5) V=XA−Y

where $\begin{array}{l} V={\left[\begin{array}{ccc} V_{1} & \ldots & V_{n} \end{array}\right]}^{T}\\ X=\left[\begin{array}{c} \begin{array}{ccc} 1 & -\cos\theta_{1} & -\sin\theta_{1} \end{array}\\ \ldots \\ \begin{array}{ccc} 1 & -\cos\theta_{n} & -\sin\theta_{n} \end{array} \end{array}\right]\\ A={\left[\begin{array}{ccc} a_{0} & a_{1} & b_{1} \end{array}\right]}^{T}\\ Y={\left[\begin{array}{ccc} d_{1} & \ldots & d_{n} \end{array}\right]}^{T} \end{array}$.

Based on the least squares principle, the optimization model can be written as

(6) $$\underset{A}{\min}f(A)={\left\|V\right\|}^{2}$$

**Figure 3:**
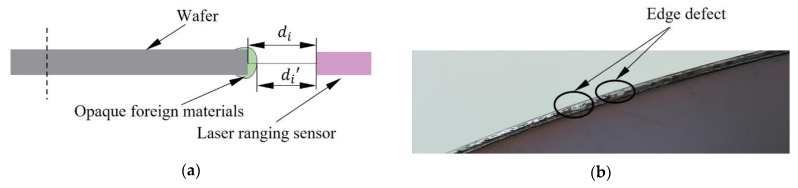
Impurities or defects at the edge of the wafer. (**a**) Opaque impurities are attached to the wafer; (**b**) Defect at the edge of the wafer.

If Formula (6) is used to evaluate the error and fit it directly, a global optimal fitting value will be obtained. However, the edge of the wafer is not a perfect circle. When we detect the edge of the wafer, we will find that the morphology defects of the wafer mainly include depressions and protrusions. For example, when photoresist is spraying on the wafer, opaque impurities often adhered to its edge, as shown in Figure 3a [20]. Additionally, there are often defects at the edge of the wafer when it is processed, as shown in Figure 3b. If the impurities or defects are detected during scanning, the distance $d_{i}$ measured by the laser ranging sensor is incorrectly detected as $d_{i}^{’}$. Additionally, $error=d_{i}-d_{i}^{’}$, which made the measured value offset from the truth value. Obviously, the global optimal results will be affected by the outliers, which will reduce the fitting accuracy.

The problem can be suppressed by adding weight $\omega_{i}$ into the error evaluation formula. If the outlier is given a lower weight, the influence of the outlier on the result can be reduced while fitting. It is important to assign a reasonable weight to each point on the edge of the circle. 

When we have a lot of edge points, and the edge points only contain random errors, then the random errors will be normally distributed. There is a probability of 68% that the error is within ±σ, and only a probability of 1% that the error is beyond ±2.6σ. Therefore, the standard deviation of the error between the fitted points and the measured points can be used as a standard for determining the weight. To obtain the initial standard deviation, we firstly let $\omega_{i}=1$, and then the FC method is used to fit the data. After the initial fitting, the initial fitting result is calculated. We can obtain the error $V_{i}$ of each point. Then, the standard deviation of the error $V_{i}$ is calculated. Finally, the point is weighted and iterated according to the standard deviation.

Moreover, while weighting, the deviation between the initial fitting point and the true value may be large. If we only weight the point with the smallest error the highest in the first fitting, the initial fitting deviation is difficult to eliminate. Therefore, the weight is designed in segments, and the corresponding formula is defined as

(7) $$\left\{\begin{array}{cc} w_{i}=1 & \left|v_{i}\right|\leq \sigma \\ w_{i}=\frac{2.6\sigma -\left|v_{i}\right|}{1.6\sigma } & \sigma <\left|v_{i}\right|\leq 2.6\sigma \\ w_{i}=0 & \left|v_{i}\right|>2.6\sigma \end{array}\right.$$

After the weight is added, the fitting error formula of all points can be rewritten as

(8) $$\underset{A}{\min}f(A)=\sum_{i=1}^{n}w_{i}(a_{0}-a_{1}\cos\theta_{i}-b_{1}\sin\theta_{i}-d_{i}{)}^{2}$$

Let w be a diagonal matrix, which consists of weights $w_{i}$. Then, f(A) can be further expressed as

(9) $$\underset{A}{\min}f(A)={\left(XA-Y\right)}^{T}w(XA-Y)$$

If we take the respect to *A* and set $f\left(A\right)=0$, *A* can be calculated by

(10) $$A={\left(X^{T}wX\right)}^{-1}X^{T}wY$$

### 2.2. Fitting the Arc of the Wafer by LSC

As shown in Figure 1, the central angle $\beta_{1}$ of the $N_{2}N_{3}$ arc is 0.49°. If n edge points are collected for each rotation of the wafer, $\frac{n\times 0.49}{360}$ points are collected in the $N_{2}N_{3}$ arc segment. It should be noted that at least three points are required to obtain the solution of the circle equation, the points collected by one rotation should satisfy $n\geq \frac{3}{\beta_{1}}\times 360$. The LSC method is used to fit the $N_{2}N_{3}$ arc segment directly. Since the $N_{2}N_{3}$ arc segment is small and few points are collected, the time spent in this step can be ignored.

While the LSC method is used to fit the center of $N_{2}N_{3}$. The equation of the circle can be written as:

(11) $${\left(x_{i}-x_{0}\right)}^{2}+{\left(y_{i}-y_{0}\right)}^{2}=R^{2}$$

where $\left(x_{0},y_{0}\right)$ is the center of the circle, ${(x}_{i},y_{i})$ is the edge point of the circle, R is the radius of the fitting circle. Additionally, it can be seen in Figure 2 that $x_{i}=(l-d_{i})\cos\theta_{i}$, $y_{i}=(l-d_{i})\sin\theta_{i}$. The error equation for fitting the circle can be written as

(12) $$V_{i}=R-R_{r}$$

where $V_{i}$ is the error value and $R_{r}$ is the true value of the circle. To solve Formula (12) directly, the square root of R needs to be calculated, which is quite complicated. Therefore, the error equation is changed to

(13) $$V_{i}=R^{2}-R_{r}{}^{2}$$

In this case, the error equation for any point can be further expressed as

(14) $$V_{i}={\left(x_{i}-x_{0}\right)}^{2}+{\left(y_{i}-y_{0}\right)}^{2}-R_{r}{}^{2}$$

The error equation for all points can be expressed as a matrix

(15) V=XA−Y

where $\begin{array}{l} V={\left[\begin{array}{ccc} V_{1} & \ldots & V_{n} \end{array}\right]}^{T}\\ X=\left[\begin{array}{c} \begin{array}{ccc} 1 & 2(l-d_{1})\cos\theta_{1} & 2(l-d_{1})\sin\theta_{1} \end{array}\\ \ldots \\ \begin{array}{ccc} 1 & 2(l-d_{n})\cos\theta_{n} & 2(l-d_{n})\sin\theta_{n} \end{array} \end{array}\right]\\ A={\left[\begin{array}{ccc} x_{0}{}^{2}+y_{0}{}^{2}-R_{r}{}^{2} & -x_{0} & -y_{0} \end{array}\right]}^{T}\\ Y={\left[\begin{array}{ccc} -{\left(l-d_{1}\right)}^{2} & \ldots & -{\left(l-d_{n}\right)}^{2} \end{array}\right]}^{T} \end{array}$.

Based on the least squares principle, the optimization model can be converted as

(16) $$\underset{A}{\min}f(A)={||V||}^{2}$$

Same as Formula (10), taking the respect to *A*, and setting $f\left(A\right)=0$, *A* can be calculated by

(17) $$A={\left(X^{T}X\right)}^{-1}X^{T}Y$$

### 2.3. Pre-Alignment Algorithm

The pre-alignment algorithm uses the WFC method and the LSC method to fit the center and the notch of the wafer, respectively. Before fitting the center of the wafer and the center of the notch, the edge points need to be classified into the notch points and the circle edge points. 

When sampling the circle edge of the wafer, the gradient of the edge is small. While the notch is scanned, the gradient will take an abrupt change. Therefore, the notch location can be initially found by calculating the first-order difference of data from the laser ranging sensor. Two peaks occur: the positive peak indicating entering the notch and the negative peak indicating leaving the notch.

When 20,000 points are obtained by the laser ranging sensor, the coordinates of the two peaks are obtained by calculating the first-order difference of the data $d_{i}$. The median point between the two peaks can be regarded as the approximate center of the $N_{2}N_{3}$ arc. The central angle $\beta_{1}$ of the $N_{2}N_{3}$ arc is 0.49°, so the $\frac{20,000}{360}\times 0.49\approx 27$ points around the approximate center are classified as $N_{2}N_{3}$ arc. The central angle $\beta_{2}$ of the notch is 1.04°, so the edge points after removing the $\frac{20,000}{360}\times 1.04\approx 57$ points around the approximate center are taken as the circle edge points.

Finally, the WFC method is used to fit the center of the circle edge points, and the LSC method is used to fit the center of the $N_{2}N_{3}$ arc points.

## 3. Simulation Analysis

All simulations were performed on the computer platform 64-bit Windows 10 operating system; Intel(R) Core (TM) i7-7700HQ CPU @ 2.80 GHz; memory 16 Gb.

A 300 mm wafer whose center coincides with the center of rotation O and the center of its notch coincides with the X axis was drawn. When the center of the wafer offset to any position and the notch deflected to any angle, the coordinates of the wafer edge can be expressed as

(18) $$\left[x^{\prime },y^{\prime }]=\left[x,y\right]\left[\begin{array}{cc} \cos\psi & -\sin\psi \\ \sin\psi & \cos\psi \end{array}\right]+[a,b\right]$$

where $\left[x,y\right]$ is the edge point of the standard wafer, ψ is the deflection angle of the expected wafer notch, [a,b] is the coordinate of the expected wafer center, and $\left[x^{\prime },y^{\prime }\right]$ is the edge point of the wafer after deflection and translation.

**Figure 4:**
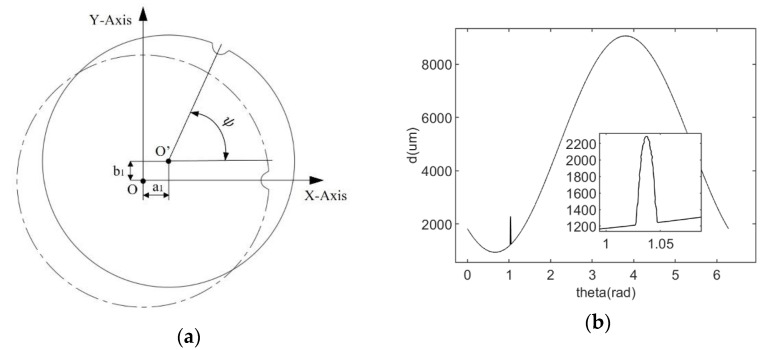
Simulation data. (**a**) The wafer after offset and deflection; (**b**) The edge points collect by rotating scanning edge detection method.

Based on Formula (18), the theoretical wafer model is obtained after deflection and translation, as shown in Figure 4a, assuming that the distance from the laser ranging sensor to the center of rotation is l=155 mm. Additionally, 20,000 edge points $\left(d_{i},\theta_{i}\right),(i\in [1,20,000])$ are selected on the edge by rotating the scanning edge detection method. For each edge point, a Gauss noise is added. Because of the impurities and defects at the edge of the wafer, some outliers are added randomly. While simulating, we can control the Gaussian noise and outlier ratio of the edge points to simulate the sampling results in complex conditions. Figure 4b shows the simulation points with σ=0.33 μm and outliers’ ratio of 5%.

### 3.1. WFC Simulation Analysis

The center fitting simulation of edge points were carried out with the LSC method and the WFC method simultaneously. We first generated 20,000 standard edge points on the wafer. The expected model was created by Formula (18), which offset and deflected from the standard wafer randomly. Then, the points were sampled by rotating the scanning edge detection method. The Gaussian noise and the outlier were added to the points. Where the standard deviation of the Gaussian noise was σϵ[0 mm,0.1 mm] and the proportion of outliers was ratio∈[0,0.1]. Each standard deviation and each outlier ratio were combined, and each algorithm was repeated 50 times under the same combination. Then, the fitting value was subtracted from the truth value each time, and the absolute value of the result was calculated. Finally, we calculated the standard deviation of the absolute fitting error for the 50 sets of points, which were generated by the same combination.

**Figure 5:**
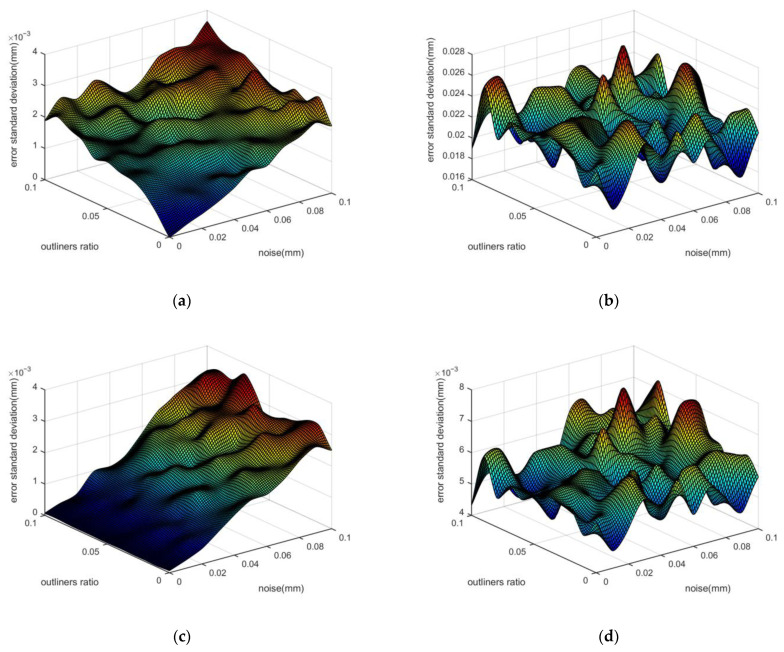
The standard deviation of fitting results of LSC method and WFC method. (**a**) Position deviation of the LSC method; (**b**) Radius deviation of the LSC method; (**c**) Position deviation of the WFC method; (**d**) Radius deviation of the WFC method.

Figure 5 shows the simulation results; where (a) and (c) show the standard deviation of the position fitting deviation of the LSC method and the WFC method, respectively. The results show that when the standard deviation of Gaussian noise is 0 and the proportion of the outliner is 0, the fitting errors of both methods are close to 0. When the proportion of outliers increases, the fitting results of the LSC method are significantly affected by the outliers, and the error increases with the increasing of the proportion of outliers. The reason for this phenomenon is that the LSC method assigns the same weight to all points to find the global optimal solution. By contrast, the WFC method can suppress the outlier well with the help of weight. When the proportion of outliers is 0, the fitting errors of both algorithms increase with the increase of the standard deviation of Gaussian noise. Additionally, the accuracy of both methods is similar. If the Gaussian noise and the proportion of outliers increase at the same time, the WFC method can suppress the influence of the outliers better than the LSC method. The consistency of the WFC method is better than that of the LSC method in different proportions of outliers.

Figure 5b and d are the standard deviations of the radius fitting of the LSC method and the WFC method, respectively. The fitting trends of both methods are similar, and the accuracy of the WFC method is about three times that of the LSC method.

**Figure 6:**
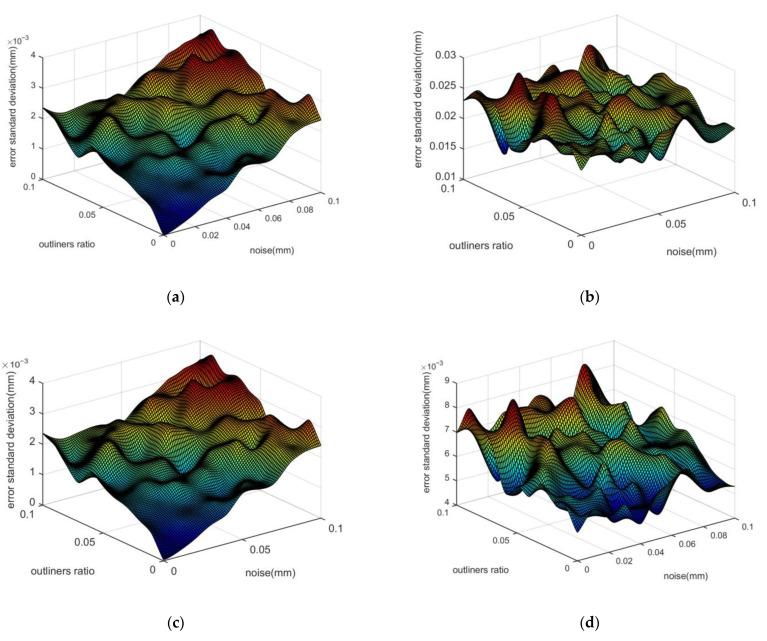
The standard deviation of fitting results of LSC method and FC method. (**a**) Position deviation of the LSC method; (**b**) Radius deviation of the LSC method; (**c**) Position deviation of the FC method; (**d**) Radius deviation of the FC method.

When the weight matrix degenerates into the identity matrix, the WFC method degenerates into the FC method, and the simulation result is shown in Figure 6. In Figure 6a,c, the standard deviation results of the position deviation of both algorithms are similar. In Figure 6b,d, the standard deviation results of the error trends of the radius are similar. Additionally, the radius fitting accuracy of the FC method is about three times that of the LSC method. Moreover, the average calculation time of the FC method is 0.249 ms, and the LSC method is 0.347 ms. The efficiency of the FC method is 28.2% higher than the LSC method.

### 3.2. Pre-Alignment Simulation and Result

The wafer pre-alignment algorithm mainly includes the classifying of notch points and circle edge points, positioning, and orientating. In simulation, the edge points were classified into the notch points and the circle edge points by gradient method. Our method uses the WFC method to fit the center of the wafer by the circle edge points, and the LSC method to fit the center of the notch by the $N_{2}N_{3}$ arc. Then, the fitting center direction is calculated. To show the advantages of our method, we compare it with traditional methods. One method is to use the CE method for positioning and the LSC method for orientating. In fact, the CE method can be used for positioning and orientation, respectively, but its performance in orientation is poor, so we use the LSC method for orientation. Additionally, the other method is to use the ME method for positioning and use the approximate median of the notch for orientating. In addition, we compared the performance of the algorithm with different numbers of points and different wafer diameters. First, standard wafers of 200 mm, 300 mm, and 450 mm with different rotation angles and the offset positions were generated by Formula (18). Then, n (from 2500 to 20,000) points were sampled by the rotating scanning edge detection method. In practice, the detection accuracy of the angle is ±3″, and the detection accuracy of the distance is ±1 μm. Therefore, according to the 3σ criterion, the standard deviation of the Gaussian noise of the angle is set to σ=1″, and the standard deviation of the Gaussian noise of the distance is set to σ=0.33 μm. 

**Figure 7:**
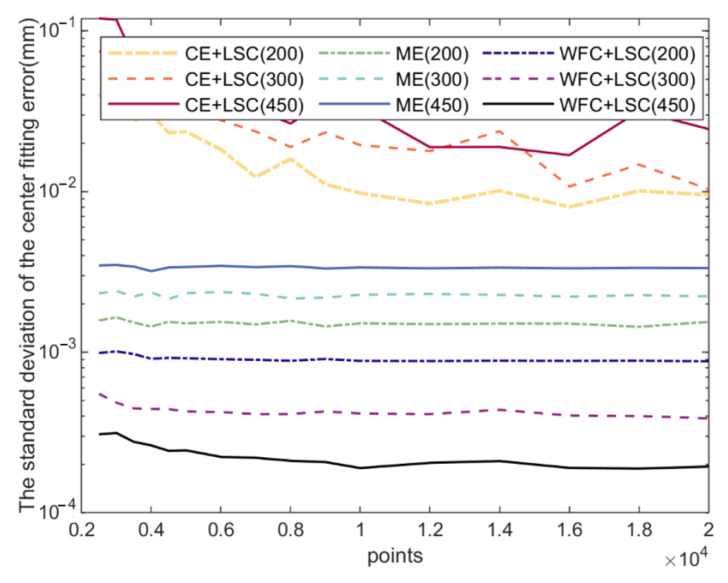
The standard deviation of the positioning error.

**Figure 8:**
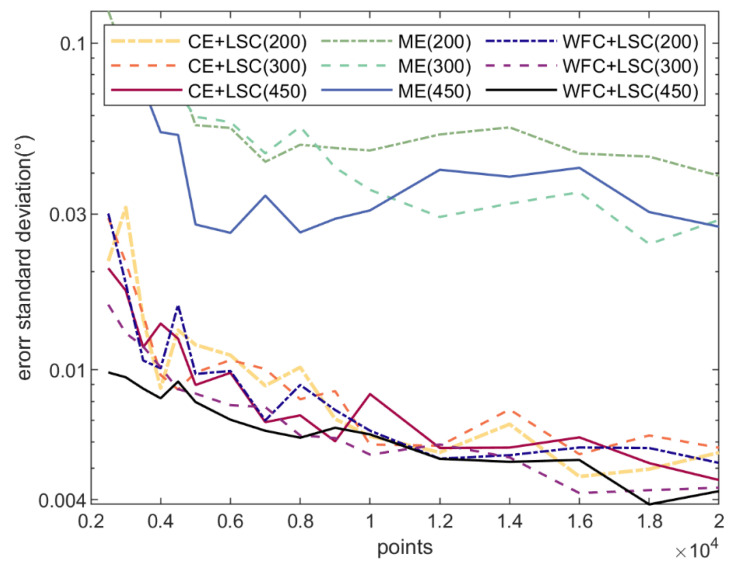
The standard deviation of the orientation error.

Figure 7 and Figure 8 show the standard deviation of the errors of fitting the center and orientation, respectively. Each wafer was sampled n (from 2500 to 20,000) points. Each combination was simulated 20 times. Finally, the standard deviation of the errors of the 20 simulation results was calculated.

The results show that the algorithm proposed in this paper is better than the traditional algorithms. The minimum n is 2500 points; the points are enough for positioning. Therefore, when fitting the center of the circle, different numbers of points have little influence on the fitting results. Additionally, the diameter of the wafer has a significant impact on the fitting results, and the larger the diameter, the higher the fitting accuracy. When fitting a 450 mm wafer, the standard deviation of error is only 0.2 μm. 

**Figure 9:**
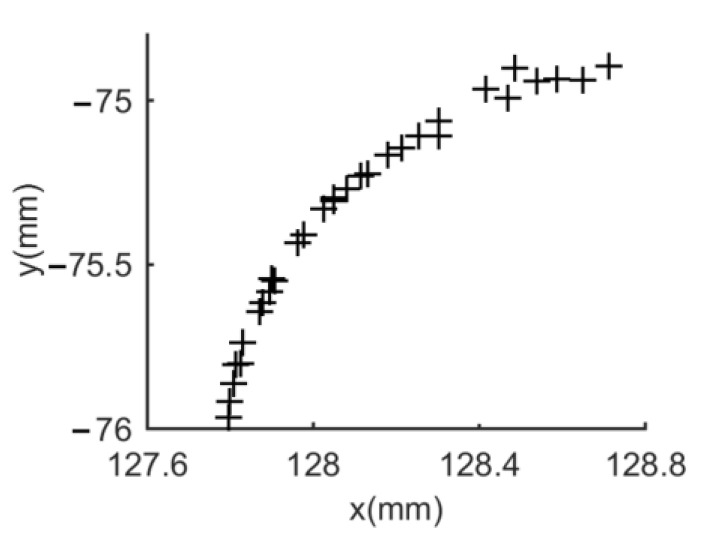
The $N_{2}N_{3}$ arc points.

During orientation, the number of sampling points for the $N_{2}N_{3}$ arc does not exceed 27($\frac{n\times 0.49}{360}$). Therefore, the number of points is small, which makes the fitting accuracy of the notch susceptible to outliners. Additionally, the number of points and the diameter of the wafer have an impact on the fitting result. The more points and the larger the wafer diameter, the higher the fitting accuracy. In particular, the CE + LSC method uses the LSC method for orientation, which is the same as the algorithm proposed in this paper. Therefore, its accuracy is higher than that of the ME method. However, the accuracy of orientation is also related to the positioning. The positioning accuracy of the CE method is much lower than that of the WFC method. Therefore, the orientation accuracy of the CE + LSC method is lower than the algorithm proposed in this paper. The ME algorithm performs poorly in both positioning and orientation. Because it cannot remove noise. Figure 9 shows the $N_{2}N_{3}$ arc points of a 300 mm wafer when sampling 20,000 points. The points on the arc are unevenly distributed due to the influence of noise. Therefore, it is difficult for the ME algorithm to obtain good fitting results.

**Figure 10:**
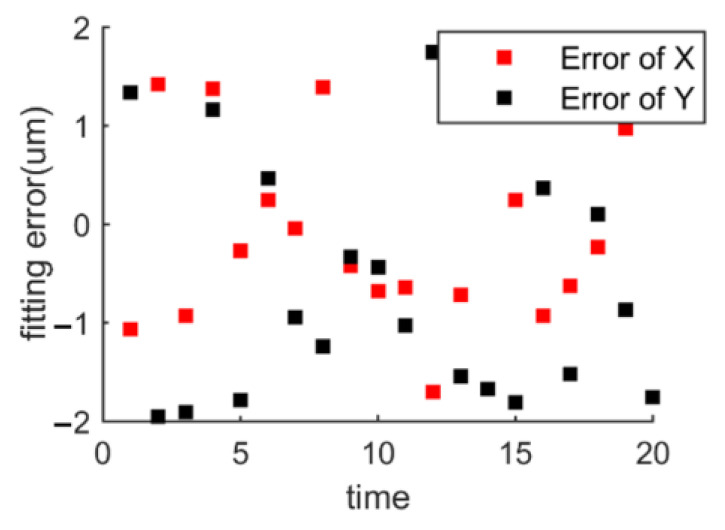
Wafer fitting error.

**Figure 11:**
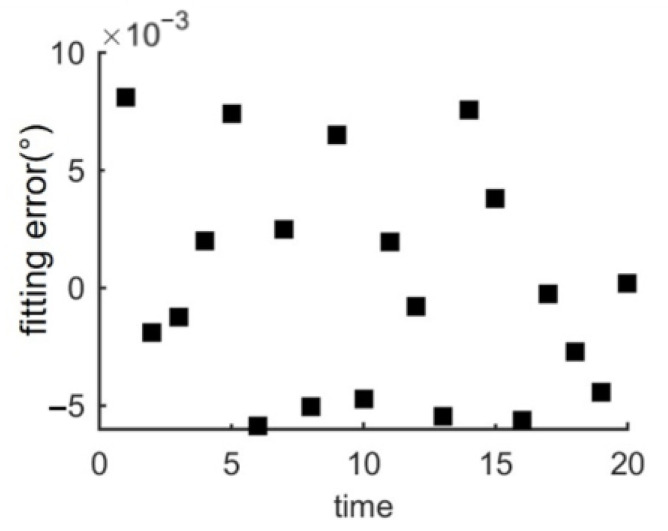
Wafer orientation error.

Finally, As shown in Figure 10 and Figure 11, when the number of sampling points n of a 300 mm wafer is 20,000, the deviations in x and y directions are ±2 μm and the orientation deviation of the wafer is ±0.01°. Additionally, the overall calculation time of the algorithm is less than 3.4 s. Moreover, the pre-alignment algorithm proposed in this paper only needs to rotate the wafer once to complete the edge point acquisition, which effectively reduces the movement time. 

## 4. Conclusions

In this paper, a new wafer pre-alignment algorithm was proposed. The WFC method is used for positioning, the LSC method is used for orientating. Simulation results showed that the positioning accuracy was ±2 μm and the orientating accuracy was 0.01°. Additionally, the overall calculation time of the algorithm was less than 3.4 s. Moreover, when fitting the center of the wafer, the WFC method can suppress the influence of outliers. When the weight of the WFC method was the identity matrix, it was degenerated into the FC method. The position fitting accuracy of the FC method in different conditions is similar to that of the LSC method, but the efficiency of the FC method is increased by 28%. In radius fitting, the WFC method always performs better than the LSC method, no matter whether the weights are degenerate or not. As a result, the pre-alignment algorithm proposed in this paper can improve the accuracy and efficiency of pre-alignment.

## Data Availability

The data presented in this study are available on request from the corresponding author.
